# HIV-1–Specific Immunodominant T-Cell Responses Drive the Dynamics of HIV-1 Recombination Following Superinfection

**DOI:** 10.3389/fimmu.2021.820628

**Published:** 2022-01-14

**Authors:** Hui Zhang, Shuang Cao, Yang Gao, Xiao Sun, Fanming Jiang, Bin Zhao, Haibo Ding, Tao Dong, Xiaoxu Han, Hong Shang

**Affiliations:** ^1^ National Health Commission (NHC) Key Laboratory of AIDS Immunology (China Medical University), National Clinical Research Center for Laboratory Medicine, The First Affiliated Hospital of China Medical University, Shenyang, China; ^2^ Key Laboratory of AIDS Immunology, Chinese Academy of Medical Sciences, Shenyang, China; ^3^ Key Laboratory of AIDS Immunology of Liaoning Province, Shenyang, China; ^4^ Clinical Laboratory, China Medical University Shengjing Hospital Nanhu Branch, Shenyang, China; ^5^ Chinese Academy of Medical Sciences Oxford Institute, Nuffield Department of Medicine, Oxford University, Oxford, United Kingdom; ^6^ Medical Research Council Human Immunology Unit, Weatherall Institute of Molecular Medicine, John Radcliffe Hospital, Oxford University, Oxford, United Kingdom

**Keywords:** HIV-1, recombination, break points, T cell responses, escape mutation

## Abstract

A series of HIV-1 CRF01_AE/CRF07_BC recombinants were previously found to have emerged gradually in a superinfected patient (patient LNA819). However, the extent to which T-cell responses influenced the development of these recombinants after superinfection is unclear. In this study, we undertook a recombination structure analysis of the *gag*, *pol*, and *nef* genes from longitudinal samples of patient LNA819. A total of 9 *pol* and 5 *nef* CRF01_AE/CRF07_BC recombinants were detected. The quasispecies makeup and the composition of the *pol* and *nef* gene recombinants changed continuously, suggestive of continuous evolution *in vivo*. T-cell responses targeting peptides of the primary strain and the recombination regions were screened. The results showed that Pol-LY10, Pol-RY9, and Nef-GL9 were the immunodominant epitopes. Pol-LY10 overlapped with the recombination breakpoints in multiple recombinants. For the LY10 epitope, escape from T-cell responses was mediated by both recombination with a CRF07_BC insertion carrying the T467E/T472V variants and T467N/T472V mutations originating in the CRF01_AE strain. In *pol* recombinants R8 and R9, the recombination breakpoints were located ~23 amino acids upstream of the RY9 epitope. The appearance of new recombination breakpoints harboring a CRF07_BC insertion carrying a R984K variant was associated with escape from RY9-specific T-cell responses. Although the Nef-GL9 epitope was located either within or 10~11 amino acids downstream of the recombination breakpoints, no variant of this epitope was observed in the *nef* recombinants. Instead, a F85V mutation originating in the CRF01_AE strain was the main immune escape mechanism. Understanding the cellular immune pressure on recombination is critical for monitoring the new circulating recombinant forms of HIV and designing epitope-based vaccines. Vaccines targeting antigens that are less likely to escape immune pressure by recombination and/or mutation are likely to be of benefit to patients with HIV-1.

## Introduction

Human immunodeficiency virus type 1 (HIV-1) is highly recombinogenic (1, 2). The infection of a cell with two or more different viral strains provides the opportunity for the generation of recombinant viruses during reverse transcription due to template switching between heterologous genomic RNAs (3, 4). Recombination facilitates the accumulation of genetic diversity in HIV-1 (5), which changes the adaptation potential of the virus, modifies its susceptibility to antiretroviral drugs, facilitates evasion from host immune responses, and affects diagnostic accuracy and vaccine design (6–11).

After superinfection, the superinfecting strain can replace the initial strain, persist with the initial strain, or exist in recombined forms in individuals (12). Several factors, such as genetic similarity (13, 14), secondary RNA structure (15, 16), and positive selection pressure by the host (17–19) or antiviral drugs (20–22), can contribute to the distribution of HIV recombination breakpoints. It has been reported that the generation of recombinants can be driven by T-cell responses (18, 23). First, the pattern of immunodominant T-cell responses often undergoes dramatic shifts after superinfection (24). One reason may be that the preexisting immune responses fail to contain the superinfecting strain due to the transmission of mutations within epitopes, resulting in a sudden increase in viral loads (VLs) (24, 25). To date, only two studies have suggested that recombination events can facilitate rapid immune escape *in vivo* (18, 23). Given the variability in interaction dynamics between HIV-1 and the host immune response and that clinical outcomes of AIDS can vary among individuals, more evidence is needed to allow the clarification of the association between the diversity of HIV-1 recombinant forms and T-cell responses.

We have previously shown that superinfection/coinfection is highly prevalent among Chinese men who have sex with men (MSM) with HIV and contributes to rapid disease progression (26). Importantly, there is recent, direct evidence to support that HIV recombinants were transmitted from a coinfected/superinfected donor (patient LNA819) to 5 putative recipients, with recombination structure analysis indicating that continuous evolution occurred *in vivo* under selection from the host (27). However, the influence of cellular immune pressure on recombination in patient LNA819 was unclear. In this study, we undertook a comprehensive analysis of the potential role of T-cell responses in HIV-1 recombination events after superinfection. We found that the quasispecies makeup and the composition of the CRF01_AE/CRF07_BC *pol* and *nef* gene recombinants among longitudinal samples of the donor changed continuously. Pol-LY10, Pol-RY9, and Nef-GL9 were the immunodominant epitopes and were either located within or flanked the regions of the recombination breakpoints. HIV-1 recombination events and/or mutations mediated escape of the dominant T-cell responses.

## Materials and Methods

### Study Participant

Patient LNA819 was recruited from a previously described (26–29) large-scale, prospective, high-risk, MSM cohort in Liaoning Province, China. Briefly, HIV-1-negative MSM were followed up every 8 weeks. A fourth-generation, enzyme-linked immunosorbent assay (ELISA) was used to screen for HIV-1 infection. ELISA-positive samples were further validated by western blotting. ELISA-negative samples were tested for HIV-1 RNA. Infection was estimated to be 14 days before the date of the RNA-positive and ELISA-negative sampling, or the midpoint of the period between the last negative and first positive results of the ELISA screening tests. Patient LNA819 reported having multiple homosexual partners and was diagnosed with HIV-1 infection on March 3, 2010, approximately 19 days post-transmission. The patient was then enrolled into an acute infection cohort and was followed up. Peripheral blood mononuclear cells (PBMCs) were isolated by Ficoll–Paque™ Plus (GE Healthcare BioScience, Stockholm, Sweden) density-gradient centrifugation at several time points of HIV infection and serial plasma samples and anticoagulated whole blood were collected. All samples were cryopreserved at −80°C until use. The study protocol was approved by the Ethics Committee of the First Affiliated Hospital of China Medical University (Shenyang, China). The patient provided written informed consent for the blood collection.

### CD4^+^ T-Cell Counts and VL Detection

CD4^+^ T cells were quantified using a FACS Calibur™ Flow Cytometry system (Becton Dickinson, Franklin Lakes, NJ, USA). Plasma HIV VLs were determined using the COBAS AmpliPrep/COBAS TaqMan HIV-1 Test (Roche, Basel, Switzerland).

### HLA Typing

DNA was extracted from anticoagulated whole blood using the QIAamp™ Blood DNA Mini Kit (Qiagen, Stanford, VA, USA) according to the manufacturer’s protocol. Two-digit HLA typing was performed with Micro SSP™ Generic HLA class I DNA typing trays using the polymerase chain reaction (PCR)-sequence-specific primer method (One Lambda, Los Angeles, CA, USA).

### HIV *gag*, *pol*, and *nef* Cloning and Sequencing

Viral RNA was extracted from longitudinal plasma samples (19, 63, 221, 300, 402, 516, 655 and 756 days post-infection [dpi]) using the QIAamp Viral RNA Mini Kit (Qiagen, Hilden, Germany). The *gag*, *pol*, and *nef* fragments were amplified using the SuperScript Polymerase One-Step RT-PCR System (Takara, Dalian, China) followed by nested PCR. The primers used are listed in **Supplementary Table 1**. For *gag* and *nef* gene amplification, the first round of PCR with outer primers was performed with the following parameters: 50°C for 30 min and 94°C for 5 min; 3 cycles of 94°C for 30 s, 50°C for 30 s, and 72°C for 2 min; 32 cycles of 94°C for 30 s, 55°C for 30 s, and 72°C for 2 min; and a final extension step at 72°C for 10 min. The conditions for the second round of PCR using the inner primers were as follows: 94°C for 5 min; 30 cycles of 94°C for 30 s, 55°C for 30 s, and 72°C for 2 min; and a final extension step at 72°C for 10 min. For *pol* gene amplification, the only difference was that extension after annealing lasted for 3 min instead of 2 min. The PCR products were confirmed through 1% agarose gel electrophoresis and purified using the QIAquick Gel Extraction Kit (Qiagen, Valencia, CA, USA). The PCR fragments were cloned using a TOPO TA cloning kit (Invitrogen, Carlsbad, CA, USA). Plasmid DNA was extracted using the QIAPrep Turbo Miniprep Kit (Qiagen, Germany) and sequenced by Huada Genomics Company (Beijing, China).

### Recombination Analysis

Individual sequence fragments were assembled using Contig Express (vector NTI suite 6.0) and then manually adjusted with BioEdit (v7.0.5). Recombination Identification Program (RIP) software was used to screen recombinants, following which the recombination structures were defined by bootscanning using Simplot (v3.5.1). The window size was 200 nt for the *pol* gene and 140 nt for the *nef* gene; the step size was 20 nt for the *pol* gene and 10 for the *nef* gene; 250 bootstrap replicates were used. Identified breakpoints were visually inspected in BioEdit.

### Synthetic Peptides

The 18-mer peptides containing 10 overlapping amino acids spanning the Gag, Pol, and Nef proteins were synthesized by Sigma–Aldrich (Milwaukee, WI, USA) (29). The peptides were designed based on the consensus sequence of 50 near-full-length CRF01_AE subtype sequences derived from patients from MSM cohort with acute HIV-1 infection in Liaoning province, China. A total of 209 overlapping peptides (OLPs) were obtained and pooled by mixing 11–16 peptides per pool in a 14×16 matrix design, yielding a total of 30 pools. The Peptides with any response in a given matrix allowed identification of the common peptide represented in the two corresponding pools. Each peptide was confirmed using PBMCs from the same time point (29). In addition, 36 HLA-matched published epitopes, or epitopes predicted by NetMHC 3.4 Server (http://www.cbs.dtu.dk/services/NetMHC/) covering the recombination area (442–1428 nt, 2613–2723 nt and 2862-2967 nt in the *pol* gene of HXB2, and 1-294 nt in the *nef* gene of HXB2), were also synthesized. The epitopes were designed based on the autologous sequences of two parental strains and were synthesized by GL Biochem (Shanghai, China) with >95% purity.

### Interferon-Gamma Enzyme-linked Immunospot Assay

Interferon-Gamma Enzyme-linked Immunospot (IFN-γ ELISPOT) assays (BD™ ELISPOT, USA) were performed as previously described (29, 30). Briefly, 96-well plates were coated with anti-IFN-γ monoclonal antibody. A total of 100,000 PBMCs were plated per well of each 96-well plate with pooled peptides or a single peptide at a final concentration of 5 μg/mL. Phytohemagglutinin (10 μg/mL) served as the positive control and medium alone as the negative control. The spots were counted using the ImmunoSpot**^®^** Analyzer (Cellular Technology, Shaker Heights, OH, USA). The results were expressed as spot-forming cells (SFCs) per 1×10^6^ PBMCs. Responses were considered positive when the number of SFCs was at least three-fold greater than the mean number of SFCs in the negative control and there were more than 50 SFCs per 1×10^6^ PBMCs. In addition, the avidity of responses to wild-type or variant epitopes was determined through serial 10-fold epitope dilutions ranging from 10 μg/mL to 10 pg/mL.

### Statistical Analysis

Data were analyzed and graphs were created using GraphPad Prism v5.0 (GraphPad Software, San Diego, CA, USA).

## Results

### The Clinical Characteristics of Patient LNA819

**Figure 1:**
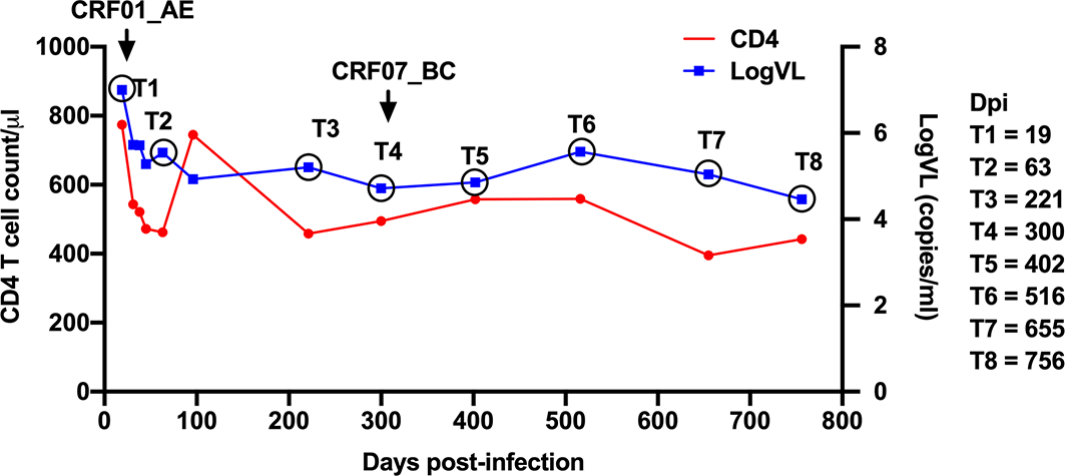
Clinical data for patient LNA819. Longitudinal clinical data for plasma viral loads (VLs) (blue line) and CD4^+^ T-cell counts (red line) after acute HIV-1 infection. Infection with the HIV-1 CRF01_AE strain was detected at 19 days post-infection (dpi) (T1); superinfection with the CRF07_BC strain was detected at 300 dpi (T4).

Patient LNA819 was diagnosed with acute HIV-1 infection in stage Fiebig IV, with indeterminate western blot profiles showing reactivity to the gp120 and p24 proteins. The estimated date of infection was approximately 19 days post-transmission based on a definite date for high-risk sexual behavior obtained from the epidemiological information. The patient’s HIV VL reached the upper limit of detection (7.00 log_10_ copies/mL), rapidly declined to 4.93 log_10_ copies/mL at 96 dpi, and then stabilized at an average of 4.97 log_10_ copies/mL over the next 22 months (96–756 dpi) (**Figure 1**). The CD4^+^ T-cell count was 774 cells/µL at 19 dpi, followed by a period of rapid decline of ~13 cells/uL per month, and was reduced to 442 cells/uL at 756 dpi (**Figure 1**). The patient was infected with an HIV-1 CRF01_AE strain at the time of diagnosis and was identified as being superinfected with a CRF07_BC strain by next-generation sequencing at 300 dpi (26). However, no sudden changes in CD4^+^ T-cell counts or VL were observed during this period. The patient did not receive antiretroviral therapy according to Chinese guidelines for AIDS diagnosis and treatment during this study. His HLA type was HLA-A*24, A*68, B*15, B*44, Cw*04, and Cw*04.

### Continuous Evolution of CRF01_AE/CRF07_BC Recombinants in the *pol* and *nef* Genes in Patient LNA819

**Figure 2:**
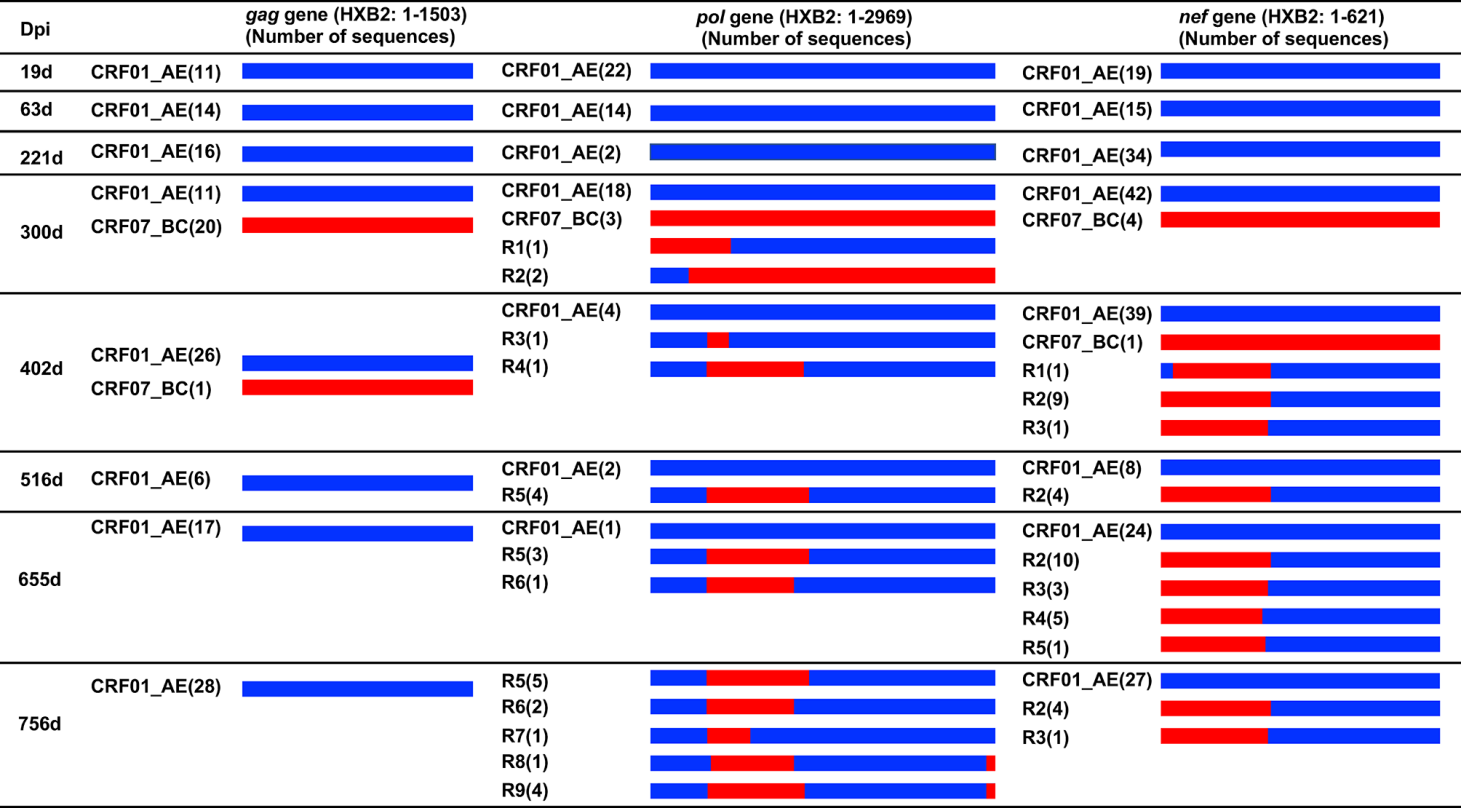
Recombination structure analysis of the *gag*, *pol* and *nef* genes from patient LNA819 at longitudinal time points. The recombination structures were defined by bootscanning using Simplot (v3.5.1) and then the breakpoints were identified and visually inspected in BioEdit. The first column represents the time points of sample collection. The second to fourth columns describe the recombination forms of the *gag*, *pol* and *nef* genes. Initial CRF01_AE strains and superinfecting CRF07_BC strains are marked as slate-blue and red, respectively. The bracketed text indicates the number of sequences of the different strains. Dpi, days post-infection.

The number of unique recombinant forms (URFs) of HIV-1 CRF01_AE/CRF07_BC was detected in patient LNA819 during the follow-up based on the recombination structure analysis of 3′ half genome sequences (27). Considering that Gag, Pol, and Nef protein-specific T-cell responses exert strong selective pressure on viral evolution, the *gag*, *pol*, *and nef* genes were amplified and sequenced at eight time points from 19 to 756 dpi. A total of 150 *gag*, 92 *pol*, and 252 *nef* cloned sequences were obtained and used for viral evolution analyses. The results showed that, in agreement with our previous studies (27), the patient was first infected with HIV-1 strain CRF01_AE, and then superinfected with a CRF07_BC strain. The latter accounted for 64.52% (20/31), 12.50% (3/24), and 8.70% (4/46) of the cloned sequences for the *gag*, *pol*, and *nef* genes at 300 dpi, respectively (**Figure 2**). The CRF01_AE strain obtained at 19 dpi and the CRF07_BC strain obtained at 300 dpi were used as putative parental sequences to perform the recombination structure analysis. The subtype F1 sequence (AF005494) was used as an outgroup. *pol* (**Supplementary Figure 1**) and *nef* (**Supplementary Figure 2**) gene recombinants were detected, but not *gag* gene recombinants. The identified breakpoints are listed in **Supplementary Table 2**.

*pol* gene recombinants were first detected at 300 dpi with a frequency of 8.75% (3/24) and represented 100% (10/10) of the sequences at 756 dpi (**Figure 2**). The CRF01_AE/CRF07_BC recombinant forms were complex. First, the composition of the recombinants and quasispecies among longitudinal samples changed continuously. A total of nine recombinant forms were identified, but none were dominant at 756 dpi. Second, with time, the number of recombinant forms identified increased from two at 300 dpi to five at 756 dpi. Only Pol-R5 and -R6 were detected at two or more sequential time points. Third, in one recombinant, the number of recombination breakpoints gradually increased from two to three. Several breakpoints, such as those at HXB2 *pol* gene positions 446–476 nt, 1420–1427 nt, and 2847–2861 nt, were detected among different recombinants at different time points. Moreover, eight of the recombinants (the exception was R2) were in a CRF01_AE backbone harboring one to two CRF07_BC insertions (**Figure 2**).

*nef* gene recombinants were first detected at 402 dpi with a frequency of 21.57% (11/51) and represented 15.63% (5/32) of all the sequences at 756 dpi (**Figure 2**). A total of five recombinant forms were identified (**Supplementary Figure 2** and **Supplementary Table 2**), all of which had a CRF01_AE backbone and one CRF07_BC fragment insertion. R1 had two breakpoints—HXB2 positions 37–42 nt and 244–252 nt—the latter of which was also present in the R2 recombinant. The other three recombinants (R3–R5) had breakpoints at HXB2 *nef* gene positions 227–252, 202–212, and 202–215 nt. In addition, several recombinants were detected at the same time point. The R2 and R3 recombinants were detected at 2 time points. The results of the recombination analysis revealed a continuous evolution of CRF01_AE/CRF07_BC recombinants *in vivo*.

### Identification of the Immunodominant Epitopes Covering the Recombination Regions

We next investigated whether recombination facilitated rapid escape from preexisting dominant T-cell responses. For this, a comprehensive IFN-γ ELISpot assay was performed to identify T-cell responses targeting peptides of the primary strain using a panel of 18-mer OLPs that mapped to the Gag, Pol, and Nef proteins of the CRF01_AE subtype. Eight peptide-specific T-cell responses were detected (**Table 1**). OLP-Pol58 and OLP-Pol59 contained the reported HLA-B*15-restricted ILKTPVHGYY (Pol 464–473, IY10) and HLA-A*24-restricted TYYDPSKDL (Pol 472–480, TL9) epitopes. Moreover, the results of epitope prediction using the NetMHC 3.4 Server supported the presence of potential B∗15-restricted LKTPVHGTYY (Pol 465–474, LY10), LKTPVHGTY (Pol 465–473, LY9), and KTPVHGTYY (Pol 466–474, KY9) epitopes in these peptides. Next, T-cell responses were detected to evaluate which epitope contributed to the responses to OLP-Pol58/OLP-Pol59. The results showed that patient LNA819 exhibited the strongest responses to the LY10 epitope, but showed no responses to the B*15-restricted IY10 and A*24-restricted TL9 epitopes (**Table 1**), suggesting that LY10 was an immunodominant epitope. In addition, the overlapping region of OLP-Pol122 and OLP-Pol123 contained the B*15-restricted RKAKIIRDY (Pol 978–986, RY9) epitope, and OLP-Nef11 contained the B*15-restricted GAFDLSFFL (Nef 94–102, GL9) epitope (**Table 1**). These results indicated that B*15-restricted LY10, RY9, and GL9 were the immunodominant epitopes.

**Table 1:** T-cell responses targeting 18-mer OLPs spanning the Gag, Pol, and Nef proteins of the HIV-1 CRF01_AE subtype after superinfection.

Name	Peptide	Position(HXB2)	Peptide-specific T-cell responses(SFCs/1×10^6^ PBMCs)	Reported/predicted epitope	Epitope-specific T-cell responses (SFCs/1×10^6^ PBMCs)
Gag13	TKEALDKIEEVQNKSQQK	Gag (97–114)	190	—	—
Pol58	ELAENREI**LKTPVHGTYY**	Pol (457–474)	280	Reported epitope: B*15—ILKEPVHGVY (IY10) A*24—VYYDPSKDL (VL9) Predicted epitope: B*15—LKTPVHGTYY (LY10) B*15—LKTPVHGTY (LY9) B*15—KTPVHGTYY (KY9)	
Pol59	**LKTPVHGTYY**DPSKDLVA	Pol (465–482)	1,310		0 0 **1,080** 50 1,250
Pol122	NSDIKVVPR**RKAKIIRDY**	Pol (969–986)	270	B*15—RKAKIIRD (RY9)	**340**
Pol123	R**RKAKIIRDY**GKQMAGDD	Pol (977–994)	70		
Nef11	MTFK**GAFDLSFFL**KEKGG	Nef (79–96)	510	B*15—GAFDLSFFL (GL9)	**6,940**
Nef14	SK**KRQEILDLWVY**NTQGF	Nef (103–120)	150	B*44—KRQEILDLWVY (KY11)	0
Nef17	YTPGPGI**RFPLCFGW**CFK	Nef (127–144)	60	A*24—RYPLTFGW (RW9)	0

OLPs, Overlapping peptides; SFCs, Spot-forming cells; PBMCs, Peripheral blood mononuclear cells. The reported epitopes within peptides are highlighted in bold form.

To further examine whether the T-cell responses targeting the epitopes within the recombination regions of the parental strains were different, the published optimal epitopes restricted by the patient’s HLA alleles mapping to the CRF07_BC insertions in the Pol R9 recombinant (the most complicated *pol* recombinant form with two CRF07_BC insertions) and the Nef R2 recombinant (containing the longest CRF07_BC insertions) were synthesized. Among the 12 epitopes assessed, 11 (except for the B*15-restricted GL9 epitope) differed between the CRF01_AE and CRF07_BC strains according to the autogenous sequence (**Table 2**). Next, a total of 23 epitopes were examined. Again, only responses to LY10, RY9, and GL9 were detected. Moreover, the magnitudes of the CRF01_AE-LY10 and CRF01_AE-RY9 epitope-specific T-cell responses were greater than those for the corresponding epitopes of the CRF07_BC strains (LY10: 2,360 *vs.* 400 SFCs/1×10^6^ PBMCs and RY9: 230 *vs.* 40 SFCs/1×10^6^ PBMCs, respectively) (**Table 2**). Based on these results, we next analyzed the effects exerted by LY10, RY9, and GL9-restricted T-cell responses on the evolution of the parental strains and recombinants.

**Table 2:** T-cell responses targeting epitopes in recombination regions.

No.	Epitope	Location(HXB2)	CRF01_AE strain	T-cell responses(SFCs/1×10^6^ PBMCs)	Superinfection strain/CRF07_BC strain	T-cell responses(SFCs/1×10^6^ PBMCs)
1	LY10	Pol (465–474)	LKTPVHGTYY	2,360	LKEPVHGVYY	400
2	RY9	Pol (978–986)	RKAKIIRDY	230	RKAKIIKDY	40
3	GL9	Nef (90–98)	GAFDLSFFL	5,200	GAFDLSFFL	5,200
4	NK9	Pol (212–220)	NTPVFAIKK	0	NTPIFAIKR	0
5	AK9	Pol (313–321)	AIFQCSMTK	0	AIFQSSMTK	0
6	KY9	Pol (328–336)	KQNPEMVIY	0	KQNPDIVIY	0
7	EW10	Pol (358–367)	EELRAHLLSW	0	EELRQHLLKW	0
8	TF9	Pol (147–155)	TKIGCTLNF	0	TQLGCTLNF	0
9	IY10	Pol (464–473)	ILKTPVHGTY	0	LLKEPVHGVY	0
10	TL9	Pol (472–480)	TYYDPSKDL	0	VYYDPSKDL	0
11	RA9	Nef (19–27)	RLRRTPPSA	0	RMRRTEPA	0
12	TL8	Nef (80–87)	TFKGAFDL	0	TFKGAVDL	0

SFCs, Spot-forming cells; PBMCs, Peripheral blood mononuclear cells. The different amino acids between the two subtype strains are underlined.

### Escape From LY10-Specific T-Cell Responses by Both Recombination and Mutation Within Epitopes

**Figure 3:**
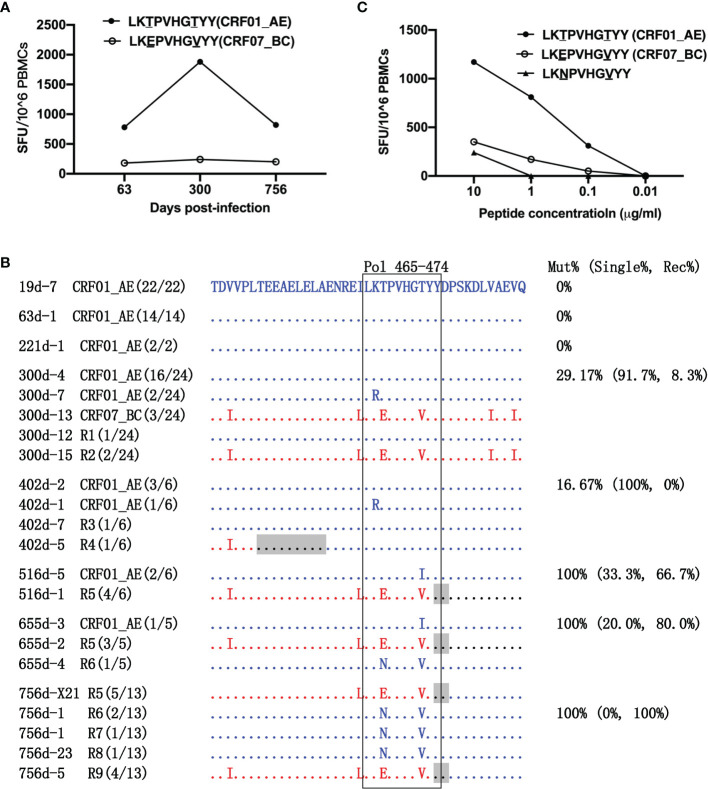
Escape from LY10 epitope-specific T-cell responses. **(A)** T-cell responses targeting the CRF01_AE-LY10 and CRF07_BC-LY10 epitopes were detected at 63, 300, and 756 days post-infection (dpi) by ELIspot. The different amino acids between the two epitopes are underlined. **(B)** The amino acid alignment of partial regions of the Pol protein covering the LY10 epitope and its flanking regions at different time points. Sequences derived from the CRF01_AE strains are highlighted in blue. Sequences derived from the CRF07_BC strains are highlighted in red. The recombination breakpoints are shaded gray. The amino acids of the LY10 epitope are boxed in black. The number in parentheses indicates the number of clones with the reported sequence. The percentages of sequences with epitope mutation (Mut%) and the percentage of single (Single%) or recombinant sequences (Rec%) are listed. **(C)** Responses to various variants within the LY10 epitope were detected using peripheral blood mononuclear cells (PBMCs) at 756 dpi.

T-cell responses targeting the variants within the LY10 epitope were examined. First, the dynamics of CRF01_AE-LY10 and CRF07_BC-LY10-specific T-cell responses were analyzed using PBMCs from 63, 300, and 756 dpi. The results showed that CRF01_AE-LY10-specific T-cell responses were first detected at 63 dpi; the magnitude of the responses had increased by more than two-fold (from 780 to 1,880 SFCs/1×10^6^ PBMCs) by 300 dpi, and then decreased to 820 SFCs/1×10^6^ PBMCs at 756 dpi. Unlike CRF01_AE-LY10, the magnitude of CRF07_BC-LY10-specific T-cell responses remained under 250 SFCs/1×10^6^ PBMCs at all three time points assessed (**Figure 3A**).

The LY10 epitope differed by 2 amino acids (positions 467 and 472 of the Pol protein) between the primary (CRF01_AE) and the superinfecting (CRF07_BC) strains. The evolution of the LY10 epitope was then analyzed (**Figure 3B**). Among the non-recombinants, the K466R and T472I variants in the CRF01_AE strains were transient. Namely, the K466R variant was first detected at 300 dpi but had disappeared at 516 dpi, while the T472I variant was first detected at 516 dpi but could no longer be detected at 756 dpi. Among the recombinants, the LY10 epitope sequences of the R2, R5, and R9 recombinants derived from the CRF07_BC strains carried the T467E/T472V variants, and, except for R2, partially overlapped with the recombination breakpoints. In addition, the LY10 epitope sequences of R1, R3, R4, R6, R7, and R8 were derived from the CRF01_AE strain, and most carried the T467N/T472V variants, except for R1, R3, and R4. Moreover, the LY10 epitope of R4 was located five amino acids downstream of the breakpoint but was located further downstream of the breakpoints in the other recombinants (R1, R3, R6, R7, and R8). The variants displayed an overall frequency of 29.17% (16/24) at 300 dpi and represented 100% (6/6) of the sequences from 516 dpi onward. The frequency of recombination-derived variants increased from 8.33% (2/24) at 300 dpi to 100% (10/10) at 756 dpi (**Figure 3B**).

In addition, patient LNA819 had a strong response to CRF01_AE-LY10 but exhibited a less efficient functional cross-recognition of CRF07_BC-LY10 and CRF01_AE-LY10 harboring the T467N/T472V variants (**Figure 3C**). These results indicated that escape from T-cell responses was mediated by both recombination with a CRF07_BC insertion carrying the T467E/T472V variants and T467N/T472V mutations originating in the CRF01_AE strain.

### The Appearance of a New Recombination Breakpoint Was Associated With Escape From RY9-Specific T-Cell Responses

**Figure 4:**
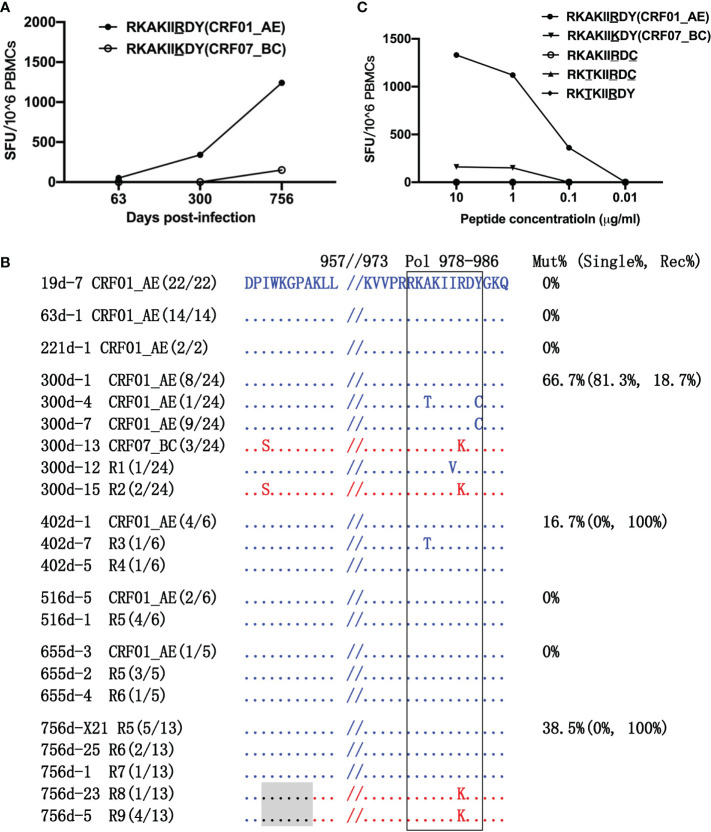
Escape from RK9 epitope-specific T-cell responses. **(A)** T-cell responses to the CRF01_AE-RK9 and CRF07_BC-RK9 epitopes were detected at 63, 300, and 756 days post-infection (dpi) by ELIspot. The different amino acids between the two subtype strains are underlined. **(B)** The amino acid alignment of partial regions of the Pol protein covering the RK9 epitope and its flanking regions at different time points. Sequences derived from the CRF01_AE strains are highlighted in blue. Those derived from the CRF07_BC strains are highlighted in red. The recombination breakpoints are shaded gray. The amino acids of the RK9 epitope are boxed in black. The number in parentheses indicates the number of clones with the reported sequence. The percentages of sequences with epitope mutation (Mut%) and the percentage of single (Single%) or recombinant sequences (Rec%) are listed. **(C)** Responses to various variants within the RK9 epitope were detected using peripheral blood mononuclear cells (PBMCs) at 756 dpi.

The amino acid at position 984 of the Pol protein differed between the CRF01_AE-RY9 and CRF07_BC-RY9 epitopes (**Figure 4A**). Responses to CRF01_AE-RY9 appeared at 300 dpi at a magnitude of 340 SFCs/1×10^6^ PBMCs. The appearance of the Y986C and A979T/Y986C variants in the CRF01_AE strains at 300 dpi, the R1 I983V variant at 300 dpi, and the R3 A979T variant at 402 dpi may have been driven by weak immune selective pressure (**Figure 4B**). Although the above-mentioned variants abrogated detectable T-cell recognition (**Figure 4C**), they disappeared during the follow-up. No variant was detected at 516 and 655 dpi. Notably, responses to CRF01_AE-RY9 markedly increased to 1,240 SFCs/1×10^6^ PBMCs at 756 dpi (**Figure 4A**); meanwhile, the CRF07_BC-RY9 epitope harboring a R984K variant, which could escape CRF01_AE-RY9-specific T-cell responses (**Figure 4C**), was observed in the R8 and R9 recombinants (**Figure 4B**). The recombination breakpoint was located ~23 amino acids upstream of the CRF07_BC-RY9 epitope. This indicated that the appearance of the new recombination breakpoint was associated with escape from RY9-specific T-cell responses. Recombination was the primary escape mechanism and accounted for 38.5% of the sequences at 756 dpi (**Figures 4B, C**).

### The F85V Variant Within the Gl9 Epitope Represented the Main Means of Immune Escape

**Figure 5:**
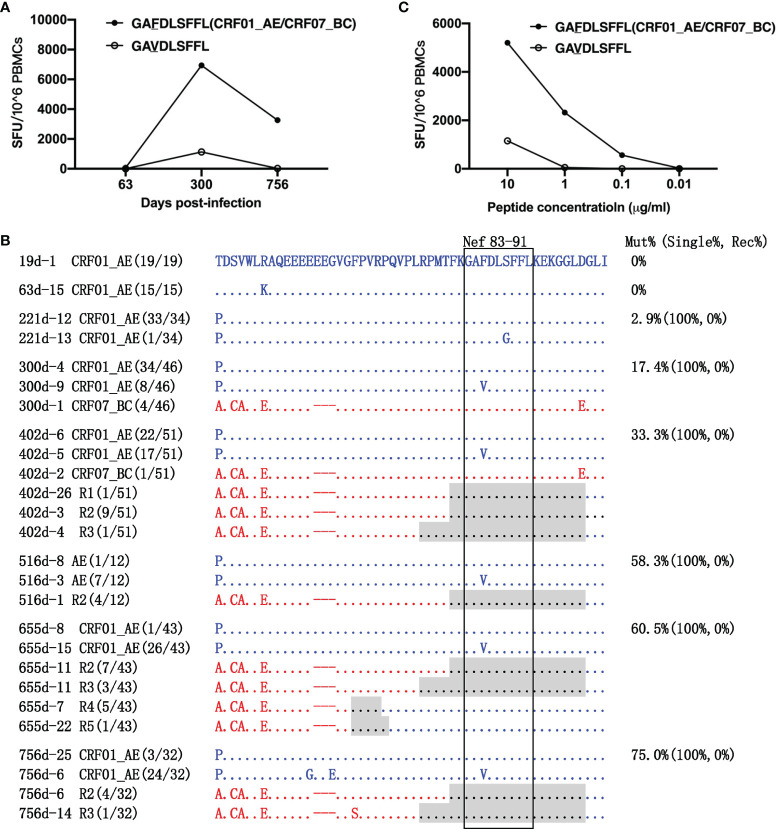
Escape from GL9 epitope-specific T-cell responses. **(A)** T-cell responses targeting the GL9 epitope and the F85V variant epitope were detected at 63, 300, and 756 days post-infection (dpi) by ELIspot. **(B)** The amino acid alignment of partial regions of the Nef protein covering the GL9 epitope and its flanking regions at different time points. Sequences derived from the CRF01_AE strains are highlighted in blue. Those derived from the CRF07_BC strains are highlighted in red. The recombination breakpoints are shaded gray. The amino acids of the GL9 epitope are boxed in black. The number in parentheses indicates the number of clones with the reported sequence. The percentages of sequences with epitope mutation (Mut%) and the percentage of single (Single%) or recombinant sequences (Rec%) are listed. **(C)** Responses to various variants within the GL9 epitope were detected using peripheral blood mononuclear cells (PBMCs) at 756 dpi.

The amino acids of the RY9 epitope from the primary CRF01_AE and superinfecting CRF07_BC strains were consistent. The GL9-specific T-cell response was strongest (6,940 SFCs/1×10^6^ PBMCs) at 300 dpi and decreased to 3,260 SFCs/1×10^6^ PBMCs at 756 dpi (**Figure 5A**). Longitudinal sequence analysis showed that a S88G variant appeared at 221 dpi with a frequency of 2.9% (1/34) and disappeared later during the follow-up. An F85V variant was first detected at 300 dpi with a frequency of 17.4% (8/46), increasing to 75.0% (24/32) at 756 dpi. The F85V mutation completely abolished T-cell recognition (**Figures 5B, C**), and thus represented the main immune escape mechanism. In addition, the GL9 epitope was located within the recombination breakpoints of R1, R2, and R3 and 10~11 amino acids downstream of those of R4 and R5. Surprisingly, however, no variant of the GL9 epitope was observed in these recombinants (**Figure 5B**).

## Discussion

HIV-1 recombination *in vivo* is poorly understood largely due to the low numbers of superinfected patients screened and characterized. We previously reported that a series of HIV-1 CRF01_AE/CRF07_BC recombinants emerged gradually in a superinfected patient (LNA819) (27). Here, we extended that study to provide unique insights into the dynamics of the development of HIV-1 recombinants and the escape from T-cell responses after superinfection. We found that responses to the Pol-LY10, Pol-RY9, and Nef-GL9 epitopes were immunodominant, and strong immune selection pressures on these epitopes may have influenced the recombination events. Indeed, the composition of the CRF01_AE/CRF07_BC recombinants in the *pol*/*nef* genes and quasispecies among longitudinal samples of the donor changed continuously under immune selection from the host, but not in the *gag* gene, where no recombination event and no strong Gag-specific T-cell responses were detected. These data indicated that escape from the immunodominant T-cell responses was achieved *via* recombination.

It has been reported that recombination facilitates HIV evasion from host immune responses, which might be associated with the transmission of escape mutations from preexisting immune responses (18, 23). In our study, two of the three immunodominant epitopes of the superinfecting virus carried escape mutations. Supportive of this hypothesis, in the setting of a strong CRF01_AE-RY9 epitope-specific T-cell response at 756 dpi, the emergence of the R984K mutation would have facilitated evasion from the immune selection pressure. Unlike the RY9 epitope, however, the escape from LY10-specific T-cell responses was complex. K466R and T472I variants was reported to diminish B*15-restricted T cell responses (31). The primary infecting strains escaped T-cell responses *via* the emergence of the K466R and T472I variants before 561 dpi. As the frequency of the recombinants increased from 8.75% at 300 dpi to 100% at 756 dpi, the rapid selection of multiple recombinant viruses that harbored the T467E/T472V variants derived from the CRF07_BC strains and the T467N/T472V variants derived from the CRF01_AE strains allowed escape from T-cell responses after 516 dpi. Therefore, the features of the escape variants in the recombinants, including mutation sites and amino acid mutation types within the epitope, differed according to the parental strain. In addition, five distinct recombinants were identified in the recombinants at 756 dpi, which suggested that the virus escaped T-cell responses through independent recombination events, which was consistent with previously published results (18, 23).

It’s worth noting that the superinfecting CRF07_BC strains were already escaped LY10 and RY9 specific T cell responses, but despite this, CRF07_BC strains did not outcompete the original CRF01_AE strains. Rather, various recombinant progeny emerged, harboring reproducible breakpoints, that recombined the “escaped” versions of LY10 and RY9 from CRF07_BC into a CRF01_AE backbone. Therefore, the recombinants might hold some additional unknown advantage beyond escape. Indeed, it has been reported that CRF33_01B, the newly emerging HIV-1 circulating recombinant form from Malaysia, acquired biological advantages in comparison to its progenitors (CRF01_AE and B), for example, the replication capacity, the rate of apoptotic cell death and the syncytia induction (32). The fitness benefits of parental and recombinant strains merit further investigation. In addition, a limitation of our study is that the sequences were obtained by bulk RT-PCR, which might generate recombinants *in vitro*. Although 44.44% (4/9) of pol recombinants and 40% (2/5) of nef recombinants were found in 12 sequences of 5’ and 19 sequences of 3’ half genomes, respectively, which were obtained from longitudinal samples by a single-genome amplification (SGA) from our previous study (27), more SGA sequences are needed to confirm the identified recombinants.

The S88G variant within the GL9 epitope was transient, appearing at 221 dpi at a low frequency (2.9%) and subsequently disappearing, which might have been associated with impaired Nef function, as reduced the down-regulation activity of CD4 and HLA-I were reported (33). In contrast, the F85V mutation, which mediated immune escape, was selected and persisted during the follow-up, and might have afforded a replication advantage. Interestingly, no variant was observed in the recombinants during the follow-up. Studies have shown that epitope-flanking amino acid variations, such as the R69K, A81G, and H87R mutations flanking the HLA-B*35-restricted VPLRPMTY epitope (VY8, Nef 74–81) (34) and the H89F change flanking the HLA-B8-restricted FLKEKGGL epitope (FL8, Nef 90–97) (35), could impair epitope generation, resulting in immune escape (36). Given that the amino acid at position 17 upstream of the GL9 epitope in all recombinants differed between the CRF01_AE and CRF07_BC strains, we speculated that recombination might impair epitope generation and endow the virus with the capacity to escape the immune response, which need to be verified in the further study.

To date, the impact of recombination on escape from T-cell responses *in vivo* has been assessed in two patients. One study noted that recombination quickly facilitated viral escape from B27-KK10 (Gag)- and Cw1-CL9 (Env)-specific T-cell responses during the chronic stage of HIV-1 infection (18). Another study, meanwhile, reported that recombination between two T/F viruses could facilitate escape from CD8^+^ T-cell responses in acute HIV infection (23). Here, we showed that two of the three identified immunodominant epitopes overlapped with the recombination breakpoints, which, in combination with previous reports (18, 23), contributes to clarifying the relationship between recombination breakpoints and epitopes. Furthermore, the different means of immune escape—recombination or mutation within epitopes—varied among epitopes, and might depend on the immunodominant response at a specific time point. This increasing complex recombination and genetic diversity may have an impact on AIDS diagnosis, treatment, prognosis, and vaccine design.

Combined, the results of the current study provide evidence that escape from the immunodominant T-cell responses targeting epitopes within or flanking recombination breakpoints was achieved *via* recombination. Mutations within epitopes and recombination events are the two most commonly detected mechanisms through which HIV-1 escapes T-cell responses in superinfected patients. Characteristics that favor a more rapid mode of escape and involve lower fitness costs are likely to favor HIV evolution. Meanwhile, understanding the pressure exerted by cellular immune responses on recombination is critical for monitoring the new circulating recombinant forms of HIV. Early initiation of antiretroviral therapy is important for preventing the generation and spread of recombinant strains. In addition, the rapid evolution and diversification of HIV-1 driven by recombination bring challenges to epitope-based vaccine design. Vaccines targeting antigens that are less likely to escape immune pressure through recombination and/or mutation are likely to be of benefit to patients with HIV-1.

## Data Availability Statement

The datasets generated/analyzed in this study are available from the corresponding authors on reasonable request.

## Ethics Statement

The studies involving human participants were reviewed and approved by the ethics committee of the First Affiliated Hospital of China Medical University. The patients/participants provided their written informed consent to participate in this study.

## Author Contributions

The study was conceived and designed by HS, XH, and TD. Data acquisition and analysis was performed by HZ, SC, YG, XS, FJ, and BZ. HD was responsible for patient recruitment. HZ wrote the first draft and HZ, SC, XH, TD, and HS contributed to the final version of the manuscript. All authors contributed to the article and approved the submitted version.

## Funding

This work was supported by the Mega Projects of National Science Research for the 13th Five-Year Plan to HS (2017ZX10201101) and the Natural Science Foundation to XH (81371787).

## Conflict of Interest

The authors declare that the research was conducted in the absence of any commercial or financial relationships that could be construed as a potential conflict of interest.

## Publisher’s Note

All claims expressed in this article are solely those of the authors and do not necessarily represent those of their affiliated organizations, or those of the publisher, the editors and the reviewers. Any product that may be evaluated in this article, or claim that may be made by its manufacturer, is not guaranteed or endorsed by the publisher.
