# Supplementary material for: HIV-1–Specific Immunodominant T-Cell Responses Drive the Dynamics of HIV-1 Recombination Following Superinfection
Source: Front Immunol. 2022 Jan 14;12:820628. doi: 10.3389/fimmu.2021.820628 (PMC8794799; doi:10.3389/fimmu.2021.820628)
Supplement: Supplementary file 1 [file DataSheet_1.docx]

Supplementary Material

**Supplemental Table 1.** Information on the primers used in cloning sequencing

| Amplicon | HXB2 position | Primers (HXB2 position) |
| --- | --- | --- |
| *gag* | 763-2400 | Outer-F: ATCTCTAGCAGTGGCGCCCGAACAG (628-648)  Outer-R: TAATGCTTTTATTTTYTCTTCTGTCAATGGC (2621-2651)  Inner-F: TGACTAGCGGAGGCTAGAAGG (763-783)  Inner-R: TTCCYCCTATCATTTTTGGTTTCC (2377-2400) |
| *pol* | 2068-5221 | Outer-F: TGGAAATGTGGA(G)AAG(A)GAA(G)GGAC (2029-2050)  Outer-R: CCTGTATGCAG(A)A(C)CCCCAATATGTT (5241-5265)  Inner-F: ACTGAGAGACAGGCTAATTTTTTAGGGA (2068-2095)  Inner-R: CTCCTAGTGGGATRTGTACTTCYGARCTTA (5192-5221) |
| *nef* | 8697-9468 | Outer-F: GTGCCTCTTCAGCTACCACCG (8513-8533)  Outer-R: AGCATCTGAGGGTTAGCCACT (9488-9508)  Inner-F: TGGACAGATAGGGTTATAGAA (8697-8717)  Inner-R: CACCTCCCCTGGAAAGTCCCC (9448-9468) |

F, forward; R, reverse.

**Supplemental Table 2.** The breakpoints of *pol* and *nef* recombinants

| *pol*  recombinants | HXB2 positions  (*pol*: 1-2969) | *nef*  recombinants | HXB2 positions  (*ne*f: 1-621) |
| --- | --- | --- | --- |
| R1 | 724-750 | R1 | 37-42 243-252 |
| R2 | 349-355 | R2 | 243-252 |
| R3 | 446-476 682-698 | R3 | 227-252 |
| R4 | 446-476 1351-1379 | R4 | 202-212 |
| R5 | 446-476 1420-1427 | R5 | 202-215 |
| R6 | 446-476 1216-1238 |  |  |
| R7 | 446-476 880-887 |  |  |
| R8 | 499-518 1216-1238 2847-2861 |  |  |
| R9 | 446-476 1420-1427 2847-2861 |  |  |

**Supplemental Figure 1. Recombination analysis of *pol* gene.** ﻿Recombination analysis *pol* gene by SimPlot (v3.5.1). The CRF01_AE strain (blue line) obtained at 19 days post-infection (dpi) and the CRF07_BC strain (red line) obtained at 300 dpi were used as putative parental sequences. The subtype F1 sequence (AF005494, gray line) was used as an outgroup. Each point plotted is the percent identity within a sliding window 200 bp wide centered on the position plotted, with a step size between points of 20 bp. Bootstrap replicate was 250.

**Supplemental Figure 2. Recombination analysis of *nef* gene.** ﻿Recombination analysis *nef* gene by SimPlot (v3.5.1). The CRF01_AE strain (blue line) obtained at 19 days post-infection (dpi) and the CRF07_BC strain (red line) obtained at 300 dpi were used as putative parental sequences. The subtype F1 sequence (AF005494, gray line) was used as an outgroup. Each point plotted is the percent identity within a sliding window 140 bp wide centered on the position plotted, with a step size between points of 10 bp. Bootstrap replicate was 250.
