# Supplementary figures and images for: HIV-1–Specific Immunodominant T-Cell Responses Drive the Dynamics of HIV-1 Recombination Following Superinfection
Source: Front Immunol. 2022 Jan 14;12:820628. doi: 10.3389/fimmu.2021.820628 (PMC8794799; doi:10.3389/fimmu.2021.820628)

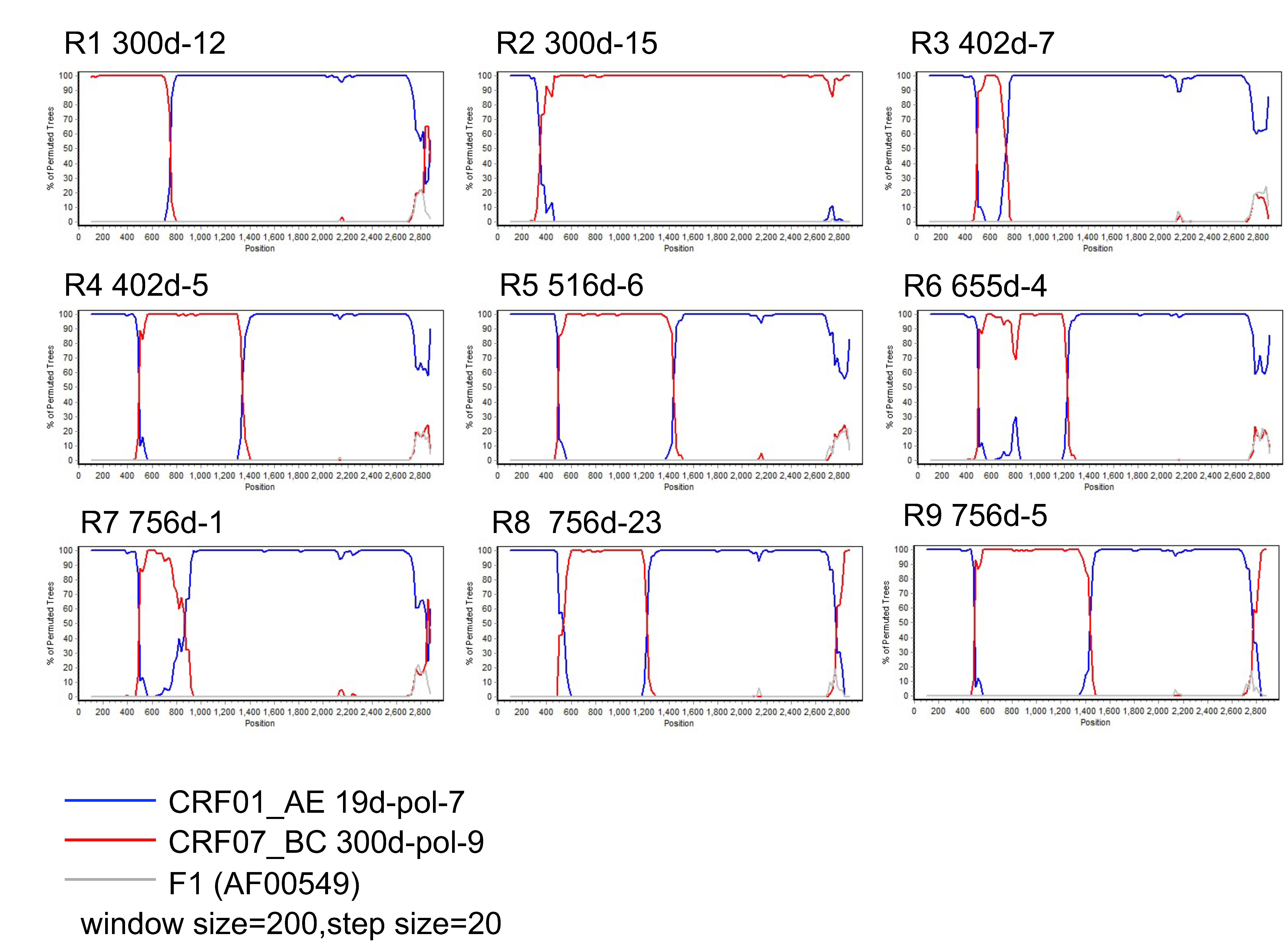

Supplement: Supplementary file 2 [file Image_1.tif]

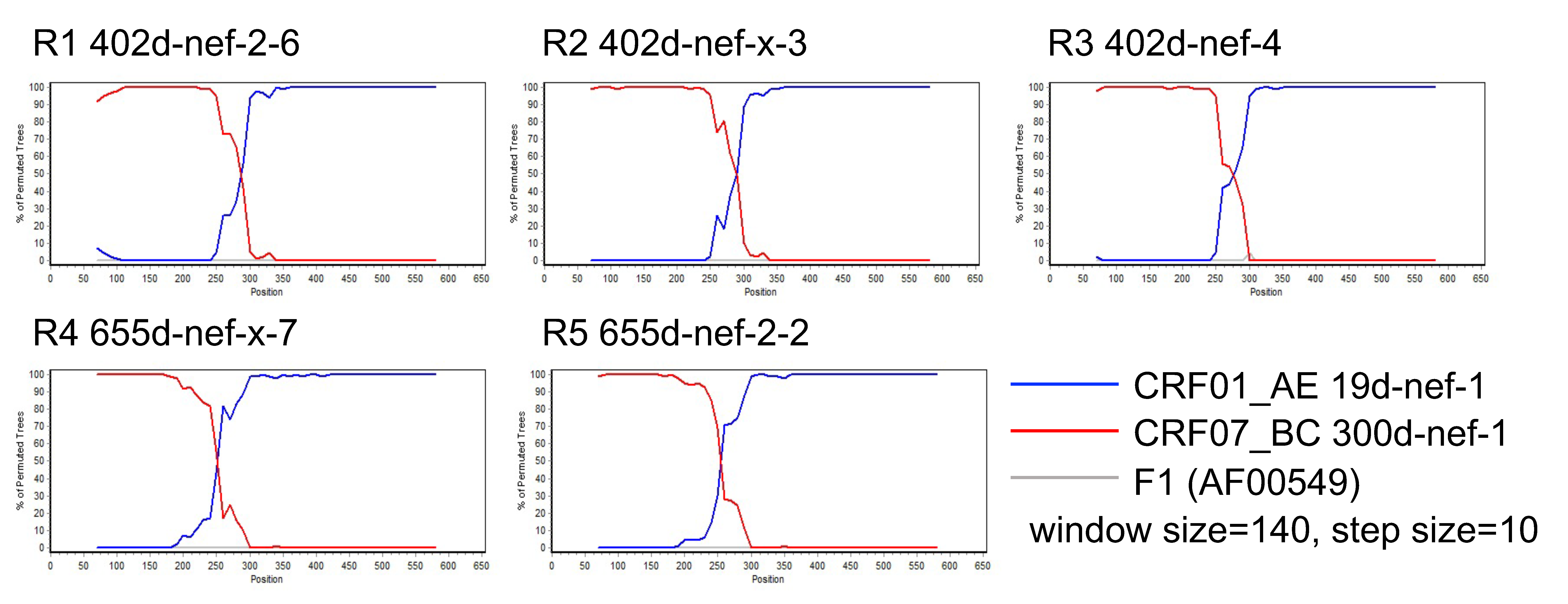

Supplement: Supplementary file 3 [file Image_2.tif]
